# Could Young Cerebrospinal Fluid Combat Glaucoma? Comment on Lee et al. Association between Optic Nerve Sheath Diameter and Lamina Cribrosa Morphology in Normal-Tension Glaucoma. *J. Clin. Med.* 2023, *12,* 360

**DOI:** 10.3390/jcm12093285

**Published:** 2023-05-05

**Authors:** Peter Wostyn

**Affiliations:** Department of Psychiatry, PC Sint-Amandus, 8730 Beernem, Belgium; wostyn.peter@skynet.be; Tel.: +32-472713719; Fax: +32-50-819720

I enjoyed reading the article by Lee et al. [1] entitled “Association between Optic Nerve Sheath Diameter and Lamina Cribrosa Morphology in Normal-Tension Glaucoma” published recently in *Journal of Clinical Medicine*. I would like to congratulate the authors for performing this cross-sectional study with findings of great importance for our understanding of the pathophysiology of normal-tension glaucoma (NTG), and I would appreciate the opportunity to make a comment on possible therapeutic implications.

The authors compared optic nerve sheath diameter (ONSD) in eyes with NTG and healthy control eyes and investigated the relationship between ONSD and lamina cribrosa (LC) morphology. They demonstrated that NTG eyes have smaller ONSDs and larger LC curvature indexes (LCCIs) than healthy control eyes, with a significant negative correlation between ONSD and LCCI. Given that NTG eyes had smaller ONSDs and that this may reflect lower cerebrospinal fluid (CSF) pressure, and given the greater degree of backward LC bowing observed in NTG eyes compared with healthy eyes, the authors concluded that lower CSF pressure may play a significant role in the pathogenesis of glaucomatous optic neuropathy in NTG. The authors also nicely reviewed additional evidence from previous studies suggesting that reduced CSF pressure may be involved in the pathogenesis of NTG.

Taken together, the above observations call for targeted research aiming to prevent or slow glaucomatous optic nerve damage via safe strategies that modulate intracranial pressure (ICP). Such treatment strategies could provide a protective effect for the optic nerve by increasing the ICP within the safe range, and thus by decreasing the trans-lamina cribrosa pressure difference, i.e., the difference between intraocular pressure and orbital subarachnoid space (SAS) pressure [2]. As discussed below, I believe research related to alterations in CSF composition could further open avenues for new approaches to the treatment of NTG. 

The optic nerve is a white matter tract of the central nervous system (CNS) that is enveloped by all three meningeal layers and is surrounded by CSF in the SAS [3,4]. Growing evidence in the literature provides strong support for the concept that not only low CSF pressure [5] but also altered CSF composition [6,7,8] within the optic nerve SAS is involved in NTG pathogenesis. Indeed, the compartmentation of the optic nerve SAS with disturbed CSF dynamics has been shown to be associated with NTG [6,7,8]. In this context, not only interventions targeting ICP but also approaches targeting CSF dynamics could be new directions in glaucoma treatment. Such interventions could improve CSF circulation around the optic nerve, leading to the enhanced removal of potentially neurotoxic waste products that accumulate in the optic nerve [2]. 

Furthermore, advanced knowledge of age-related changes in CSF composition is essential to better understand age-associated neurodegenerative diseases and might further open up new therapeutic strategies for NTG. CSF contains a complex mix of substances, including vitamins, peptides, nucleosides and growth factors, that are crucial for CNS health [9]. However, CSF protein composition changes dramatically with age [10]. For example, there is a decrease in growth factors such as brain-derived neurotrophic factor (BDNF) [10]. Studies revealed that BDNF plays an essential role in maintaining the health of retinal ganglion cells and that the deprivation of BDNF leads to the induction of their apoptosis [11,12]. The reduced retrograde axonal transport of BDNF from the brain to the retina has been suggested as a likely mechanism of glaucomatous optic neuropathy [11,12]. Intriguingly, a new study conducted by Iram et al. [10] discovered that the intracerebroventricular infusion of young CSF into aged mice has rejuvenating effects on the brain. It was found that infusing young CSF directly into aged brains improves memory function, which occurs along with an increase in oligodendrocyte progenitor cell proliferation and hippocampal myelination. Fibroblast growth factor 17, whose levels decrease with age in human CSF, was identified as a major component of the rejuvenating effects of young CSF. Given the altered CSF protein composition in aging [10], and given that NTG is a neurodegenerative disease associated with increased age, the question is whether such age-related changes in the composition of CSF within the optic nerve SAS contribute to the pathogenesis of glaucomatous optic neuropathy. If confirmed, it would be worthwhile to further explore whether the intrathecal administration (or administration via other routes such as topical and intravitreal) of factors present in young CSF might be a therapeutic strategy for glaucoma, given the significant number of patients experiencing this devastating disease for whom existing treatment options are ineffective. Intrathecal infusion pumps are already widely used for the management of chronic pain (morphine pump) and spasticity (baclofen pump) [13,14]. If the intrathecal administration of such CSF factors was proven to be effective in treating glaucoma, this new treatment, if enriched with brain rejuvenating factors, could also protect against age-related cognitive decline, as reported by Iram et al. [10]. The latter is especially important for patients with NTG, who have been shown to have a significantly higher risk of developing Alzheimer’s disease [15].
